# Delta opioid receptor agonists activate PI3K–mTORC1 signaling in parvalbumin-positive interneurons in mouse infralimbic prefrontal cortex to exert acute antidepressant-like effects

**DOI:** 10.1038/s41380-024-02814-z

**Published:** 2024-12-06

**Authors:** Toshinori Yoshioka, Daisuke Yamada, Akari Hagiwara, Keita Kajino, Keita Iio, Tsuyoshi Saitoh, Hiroshi Nagase, Akiyoshi Saitoh

**Affiliations:** 1 Laboratory of Pharmacology, Faculty of Pharmaceutical Sciences, Tokyo University of Science, Chiba, Japan; 2 Department of Applied Biological Science, Faculty of Science and Technology, Tokyo University of Science, Chiba, Japan; 3 International Institute for Integrative Sleep Medicine (WPI-IIIS), University of Tsukuba, Ibaraki, Japan

**Keywords:** Neuroscience, Molecular biology

## Abstract

The delta opioid receptor (DOP) is a promising target for novel antidepressants due to its potential for rapid action with minimal adverse effects; however, the functional mechanism underlying acute antidepressant actions remains elusive. We report that subcutaneous injection of the selective DOP agonist KNT-127 reduced immobility in the forced swimming test, and that this antidepressant-like response was reversed by intracerebroventricular injection of the selective mechanistic (or mammalian) target of rapamycin (mTOR) inhibitor rapamycin or the phosphatidylinositol-3 kinase (PI3K) inhibitor LY294002. KNT-127 also alleviated social avoidance and reduced sucrose consumption (anhedonia) among chronic vicarious social defeat stress model mice, which were similarly reversed by PI3K and mTOR inhibitors. In addition, KNT-127 increased phosphorylation levels of the mTOR signaling-related proteins Akt and p70S6 kinase in medial prefrontal cortex as revealed by immunoblotting. In the forced swimming test, a microinfusion of KNT-127 and another DOP agonist SNC80 in the infralimbic prefrontal cortex (IL-PFC) attenuated the immobility, which were blocked by rapamycin and LY294002. Perfusion of KNT-127 onto IL-PFC slices increased miniature excitatory postsynaptic current frequency and reduced miniature inhibitory postsynaptic current frequency in pyramidal neurons as measured by whole-cell patch-clamping, and both responses were reversed by rapamycin. Imaging of brain slices from transgenic mice with DOP-promoter-driven green fluorescent protein revealed that most DOPs were expressed in parvalbumin-positive interneurons in the IL-PFC. These findings suggest that DOP agonists exert antidepressant-like actions by suppressing GABA release from parvalbumin-positive interneurons *via* the PI3K–Akt–mTORC1–p70S6 kinase pathway, thereby enhancing IL-PFC pyramidal neuron excitation.

## Introduction

It is widely accepted that the Gi/o protein-coupled delta opioid receptor (DOP) contributes to mood regulation and thus is a promising target for the treatment of mood disorders [1]. For example, mice deficient in DOPs exhibit depressive- and anxiety-like behaviors [2], while inhibition of enkephalinase, the enzyme that degrades the endogenous DOP ligand enkephalin, produces antidepressant-like behavior in rats [3]. We previously reported that selective DOP agonists such as SNC80 and KNT-127 exert antidepressant- and anxiolytic-like effects in rodents [4–6]. In particular, KNT-127 exhibits high DOP selectivity and agonistic activity compared with other prototype DOP agonists [7]. In addition, KNT-127 demonstrates high blood–brain barrier permeability and potentially acts as a biased ligand that mainly activates cyclic adenosine monophosphate (cAMP) signaling with lower beta-arrestin signaling activation [8]. Therefore, KNT-127 is considered one of the most suitable compounds for clarifying the functional mechanisms underlying DOP effects on cAMP signaling and depression-like behaviors. We have demonstrated that KNT-127 exerts antidepressant-like effects in mice subjected to a forced swimming test (FST) and in olfactory bulbectomized rats [5, 6, 9]. In rodents, KNT-127 exerts anxiolytic-like effects in the elevated plus-maze, open field, and light–dark tests [10–12] as well as anti-hyperalgesic effects against inflammatory pain [6] and antimigraine-like effects in a migraine mouse model [13]. To the best of our knowledge, KNT-127 has no adverse effects such as memory impairment, addiction, hyperlocomotion, motor coordination deficits, ethanol interactions, convulsions, and digestive symptoms [5, 6, 8, 10, 14]. Thus, we propose that selective DOP activation is a promising therapeutic strategy for the treatment of emotional disorders. Indeed, we and others have conducted or are currently conducting clinical trials of DOP agonists as antidepressants [14]. However, the functional mechanisms underlying these effects of DOP agonists must be clarified to permit broader clinical testing and ultimate regulatory approval for this therapeutic strategy.

The mechanistic (or mammalian) target of rapamycin (mTOR) is implicated in the molecular and cellular mechanisms underlying antidepressant-like actions [15–17], including induction of synaptogenesis in the medial prefrontal cortex (mPFC) and accelerated neurogenesis in the hippocampus associated with the rapid and sustained antidepressant-like effect of ketamine [18, 19]. In addition, chronic fluoxetine treatment has been shown to relieve chronic unpredictable mild stress-induced behavioral deficits through mTOR signaling in the hippocampus [20].

Multiple studies have reported that mood disorders are associated with changes in glutamatergic neurotransmission [21]. For instance, the NMDA receptor antagonist ketamine has been shown to exert antidepressant-like effects in preclinical and clinical studies [18, 22]. Furthermore, abnormalities in the monoaminergic system, which are often observed in depressive and anxiety states, also alter glutamatergic system activity. In light of these findings, the classical “monoamine hypothesis” has been supplanted by a “glutamate hypothesis” [23]. In fact, clinical studies using magnetic resonance spectroscopy and positron emission tomography have revealed substantial alterations in glutamate as well as gamma-aminobutyric acid (GABA) concentrations in patients with major depressive disorder (MDD), suggesting that depression is related to an imbalance between glutamate-mediated excitatory and GABA-mediated inhibitory neurotransmission [24].

In the present study, we attempted to elucidate the cellular mechanisms underlying the antidepressant-like effects of DOP agonists. Accordingly, we aimed to determine (1) whether mTOR signal transduction is involved in the antidepressant-like effect, (2) the specific brain region(s) in which the effect is exerted, (3) the relationship between the effect and glutamatergic and/or GABAergic neuronal responses, and (4) the cell-type specificity of DOP expression.

## Materials and methods

### Animals

Male ICR mice, aged 4–5 weeks, were obtained from CLEA Japan. Male and female C57BL/6 J mice, aged 5 weeks, and male “aggressor” ICR retired breeder mice were obtained from Sankyo Labo Service Corporation Inc. (Tokyo, Japan). Male and female DOP-eGFP mice [25] were obtained from Jackson Laboratory Japan Inc. (Kanagawa, Japan), bred through natural mating, and genotyped by PCR to obtain the homozygotes used for experiments. Four to six mice were randomly housed per cage (225 × 338 × 140 mm) under controlled air temperature and pressure under a 12 h/12 h light/dark cycle (lights on from 08:00 to 20:00) with *ad libitum* access to food and water. Mice were acclimatized to the breeding room for at least 5 days before experiments. All behavioral tests were performed during the light cycle. Animal care and experimental protocols were conducted in accordance with the guidelines set forth by the animal welfare committees at Tokyo University of Science (approval numbers Y19032, Y20020, Y21002, and Y22014).

### Drug administration

For systemic drug administration, 10 mL/kg of the indicated drug solution was injected subcutaneously (s.c.) 30 min before behavioral testing or sacrifice for biochemical analysis unless otherwise stated. For drugs administered intracerebroventricularly (i.c.v.), each 5 μL solution was injected directly into the lateral ventricle using a double-stage needle under inhalational isoflurane anesthesia 60 min before behavioral testing or sacrifice for biochemical analysis, or 30 min before sacrifice for electrophysiological experiments. For local infusion, guide cannula(s) was implanted bilaterally into the target regions 7 days before the test to allow for recovery. Subsequently, the mice were infused with 0.2 μL/side of drug solution at 0.2 μL/min using a microsyringe pump. For prelimbic prefrontal cortex (PL-PFC) infusion, 26-gauge dual guide cannula (0.8 mm interval; P1 Technologies, Roanoke, VA, USA) was placed at +1.9 mm [anterior/posterior (AP)], ±0.4 mm [medial/lateral (ML)], and −2.4 mm [dorsal/ventral (DV)] from the bregma [26]. For infralimbic prefrontal cortex (IL-PFC) infusion, 25-gauge guide cannulas (Eicom, Kyoto, Japan) were angled at 30° to the sagittal plane to avoid damaging the PL-PFC and placed at +1.7 mm (AP), ±0.4 mm (ML), and −3.0 mm (DV) from the bregma. After each test, the injection (i.c.v.) or cannula implantation sites were verified by serial coronal sectioning of the brain. Data from animals in which the position was inappropriate were discarded.

KNT-127 was synthesized according to the previous report [7]. To improve the solubility in aqueous solution for in vivo assays, KNT-127 was converted into its hydrochloric acid salt (KNT-127･HCl). The purity of the sample was assessed with elemental analysis (Anal Calcd for C_24_H_24_N_2_O_2_·HCl·3.6H_2_O: C, 56.50; H,6.56; N, 5.49. Found: C, 56.52; H,6.70; N, 5.66). KNT-127 was dissolved at 1 mg/mL in saline for systemic administration or 1 mM in phosphate-buffered saline (PBS) for local infusion. The DOP agonist SNC80 and DOP inhibitor naltrindole were purchased from Merck KGaA (Darmstadt, Germany) and dissolved at 1 mM and 2 mM in PBS, respectively. Ketamine (Daiichi Sankyo Co. Ltd., Tokyo, Japan) was diluted at 2 mg/mL in saline. Rapamycin (LC Laboratories, Woburn, MA, USA) was dissolved at 0.04 mM in 5% DMSO/saline, the PI3K inhibitor LY294002 (FUJIFILM Wako Pure Chemical, Osaka, Japan) at 4 mM in 10% DMSO/saline, and the MEK inhibitor U0126 (FUJIFILM Wako Pure Chemical) at 4 mM in 4% DMSO/saline. Vehicle controls were administrated the same formulation and volume of solution without the active compound.

### Forced swimming test (FST)

The FST was conducted as previously reported with minor modifications (see Supplemental Information for details) [5].

### Locomotor activity measurement

The measurement of locomotor activity was performed as previously reported with minor modifications (see Supplemental Information for details) [27].

### Chronic vicarious social defeat stress paradigm and behavioral tests

The chronic vicarious social defeat stress (cVSDS) model was established, and the social interaction test (SIT) was conducted as previously reported (see Supplemental Information for details) [28–31]. In the sucrose preference test (SPT), singly housed cVSDS mice were given the choice of a water bottle containing an aqueous solution of 1% sucrose and an identical bottle containing only water on day 36 after the first vicarious defeat session, with the starting positions counterbalanced across tested mice. The positions of the two bottles were then switched on day 37, followed by removal for 22 h on day 38 to induce mild dehydration. On day 39, the bottles were placed back into the cage (with position randomized among mice), and the volumes consumed from each bottle measured after 2 h. Sucrose preference (%) was calculated as sucrose solution consumption divided by total water + sucrose consumption.

### Western blotting

Target brain regions were dissected, rapidly frozen in liquid nitrogen, and stored at −80 °C until use. Samples were homogenized in a cell lysis buffer (1% Triton X-100, 0.1% SDS, 50 mM Tris-HCl, 150 mM NaCl, 2 mM K_2_EDTA, phosphatase inhibitors, and protease inhibitors) and centrifuged for 15 min at 2500 × *g* and 4 °C. The total protein concentration in the supernatant was measured using a BCA Protein Assay Kit (Thermo Fisher Scientific, Waltham, MA, USA). Supernatant proteins (6 μg per gel lane) were then separated by 7.5–10% SDS-PAGE and subsequently transferred to PVDF membranes. The membranes were blocked by incubation in TBS-T (50 mM Tris, 138 mM NaCl, 2.7 mM KCl, and 0.1% Tween-20; pH 7.4) containing 5% skim milk or bovine serum albumin for 1 h at room temperature, and subsequently incubated with TBS-T containing the following primary antibodies (all from Cell Signaling Technology, Danvers, MA, USA) for 1 h at room temperature: p-Akt (1:1000; #9271), total Akt (1:1000; #9272), p-extracellular signal-regulated kinase (ERK; 1:1000; #9101), total ERK (1:1000; #9102), p-p70S6 kinase (p70S6K; 1:1000; #9234), and total p70S6K (1:1000; #2708). After rinsing with TBS-T, the membrane was incubated with horseradish peroxidase-conjugated anti-rabbit secondary antibody (1:5000; ab6721, Abcam, Cambridge, UK) for 1 h at room temperature. Target protein bands were detected using enhanced chemiluminescence reagent and captured using the LAS-4000 system (FUJIFILM, Tokyo, Japan). Protein expression levels were quantified densitometrically using ImageJ (NIH, Bethesda, MD, USA). For phosphorylated proteins, densitometry values were normalized to total protein.

### Acute slice preparation and whole-cell patch-clamp recordings

Brain slice preparation and patch-clamp recordings were performed as previously reported with minor modifications (see Supplemental Information for details) [32, 33].

### Analysis of miniature excitatory postsynaptic currents (mEPSCs)

Patch electrodes (resistance 4–6 MΩ) were filled with a solution containing 132 mM K-gluconate, 3 mM KCl, 10 mM HEPES, 0.5 mM EGTA, 1 mM MgCl_2_, 12 mM sodium phosphocreatine, 3 mM ATP magnesium salt, and 0.5 mM GTP (adjusted to pH 7.4 using KOH). Postsynaptic currents were measured in voltage-clamp mode at a clamped-estimated resting potential of −65 mV. Immediately after establishing the whole-cell configuration, a series of depolarizing rectangular current steps (750 ms; 20 pA increments; interpulse interval of 3 s) was administered to verify action potential firing with spike-frequency accommodation characteristic of healthy pyramidal neurons. The presence of spontaneous postsynaptic currents was also verified. Subsequently, slices were perfused with tetrodotoxin (TTX; 1 μM) for 5 min to block voltage-gated sodium channels, and miniature postsynaptic currents were recorded for 1 min (baseline). KNT-127 (3 or 10 μM) was then perfused for 15 min, and the currents were recorded again for 1 min. All current traces were analyzed using Mini Analysis software v.6.0.7 (Synaptosoft, Decatur, GA, USA) with threshold amplitude for detection set at 7.5 pA. The times required for 10–90% rise and 37% decay were also measured for comparison of current kinetics between conditions (before and after the application of KNT-127). If the frequency was changed to zero Hz after the bath application of KNT-127, the amplitude, rise time, and decay time were excluded from the data.

### Analysis of miniature inhibitory postsynaptic currents (mIPSCs)

mIPSCs were recorded using a pipette solution containing 105 mM K-gluconate, 30 mM KCl, 10 mM HEPES, 0.5 mM EGTA, 1 mM MgCl_2_, 12 mM sodium phosphocreatine, 3 mM ATP magnesium salt, and 0.5 mM GTP. The membrane potential was clamped at −70 mV, and in addition to TTX, slices were perfused with both CNQX (20 μM) and d-AP5 (50 μM) to block AMPA and NMDA receptor currents. All other procedures were the same as described for mEPSC recording.

### Immunohistochemistry

Immunostaining was conducted as previously reported with minor modifications (see Supplemental Information for details) [6, 25, 31]. The emissions from fluorescence-positive cells were quantified using a confocal microscope (TCS SP8, Leica Microsystems) at ×630. First, the region of interest (the IL-PFC) in each section was determined by overlaying the fluorescence image on a bright-field image and marking the corresponding area with reference to the mouse brain atlas [26]. Fluorescence signals were acquired by line-scanning of three colors (ultraviolet, green, and red) with altering Z axis. Laser intensity and detected emission band width were then carefully adjusted to prevent cross-excitation and bleed-through. The numbers of DOP- and/or PV-positive neurons in the IL-PFC (all 21 sections per mouse) were counted manually from emission profiles by a well-trained observer. A DOP-positive neuron was defined as a NeuN-labeled cell with eGFP signal emission from more than half of the cell soma surface, and a PV-positive neuron with PV-linked fluorescence emission from the entire cell soma.

### Statistical analysis

The sample size required for each experiment was determined using G*power version 3.1.9.6 based on a significance threshold of 0.05, statistical power of 0.80, and effect sizes from previous reports [5, 18, 30, 32]. All data are acquired from at least two independent experiments. Each experiment was conducted by investigators blinded to animal group and/or drug administration information. Data are presented as individual data points and the mean ± standard error of the mean (S.E.M.). All statistical analyses were performed using GraphPad Prism7 (GraphPad Software, San Diego, CA, USA). The Shapiro-Wilk test was utilized to assess normality. Two groups were compared by *t*-test, and all other comparisons by analysis of variance (ANOVA) followed by *post-hoc* tests for pairwise comparisons (see the figure legends for details). Statistical significance (two-sided) is indicated by ^*/#^*p* < 0.05 and ^**/##^*p* < 0.01.

## Results

### KNT-127 exerts antidepressant-like effects through DOPs

**Fig. 1:**
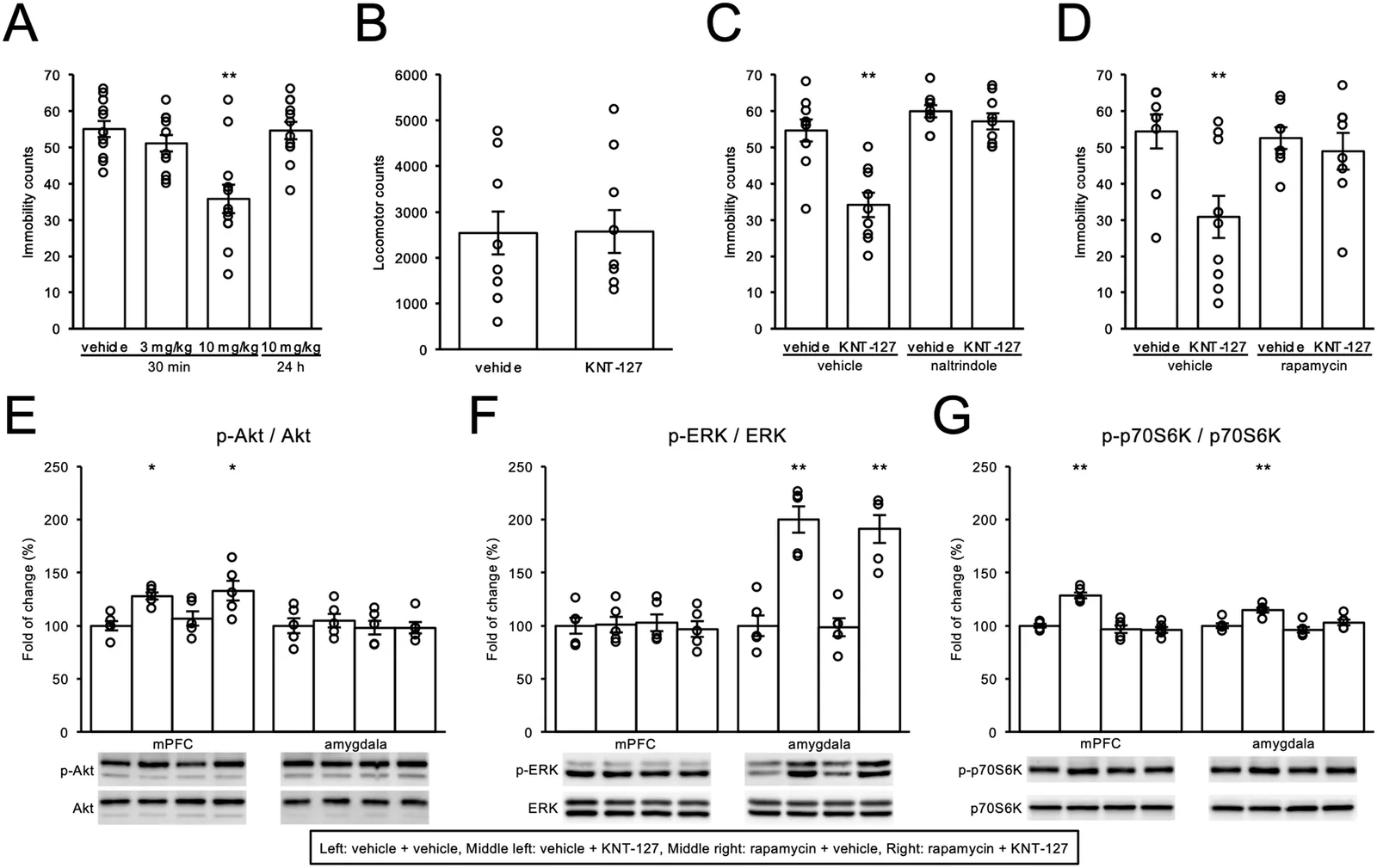
KNT-127 suppresses depression-like behavior (immobility) in mice during the forced swimming test (FST) by activating the delta opioid receptor (DOP)–Akt–mechanistic target of rapamycin complex 1 (mTORC1)–p70S6 kinase (p70S6K) signaling pathway in medial prefrontal cortex (mPFC). **A** Dose- and time-dependent immobility counts of mice subcutaneously (s.c.) administered KNT-127 in the FST. **B** Total locomotor activity counts in an acrylic cage over 40 min following administration of KNT-127 (10 mg/kg, s.c.). **C** The DOP inhibitor naltrindole [10 nmol, intracerebroventricularly (i.c.v.), 60 min before the test] inhibits the antidepressant-like effect of KNT-127 (10 mg/kg, s.c., 30 min before the test) in the FST. **D** The mTOR inhibitor rapamycin (0.2 nmol, i.c.v., 60 min before the test) abolishes the effect of KNT-127 in the FST. **E–G** Phosphorylation levels of signaling proteins upstream **E, F** and downstream **G** of mTORC1 in the mPFC and amygdala normalized to the vehicle-vehicle group as estimated by Western blotting and densitometry (typical blots shown below each panel). **E** Ratio of p-Akt to total Akt. **F** Ratio of extracellular signal-regulated kinase (ERK) to total ERK. **G** Ratio of p-p70S6K to total p70S6K. Data for vehicle-pretreated groups were acquired from distinct experiments **C, D**. Data are presented as individual data points and the mean ± S.E.M. Statistical analyses were performed as follows: one-way factorial ANOVA followed by Dunnett’s *post-hoc* test compared with the vehicle-vehicle group **A**; Student’s *t*-test **B**; two-way factorial ANOVA followed by Dunnett’s *post-hoc* test compared with the vehicle-vehicle group **C**–**G**. ^*^p < 0.05 and ^**^p < 0.01. **A–D** *n* = 8–12 mice per group; **E–G** *n* = 5 mice per group. [**A** *F*_(3, 44)_ = 10.59, *p* < 0.01. **C** Main effect of agonist: *F*_(1, 33)_ = 18.71, *p* < 0.01; main effect of inhibitor: *F*_(1, 33)_ = 27.66, *p* < 0.01; interaction effect: *F*_(1, 33)_ = 10.84, *p* < 0.01. **D** Main effect of agonist: *F*_(1, 31)_ = 7.593, *p* < 0.01; main effect of inhibitor: *F*_(1, 31)_ = 2.716, nonsignificant; interaction effect: *F*_(1, 31)_ = 4.080, nonsignificant. **E** Main effect of agonist: *F*_(1, 16)_ = 14.58, *p* < 0.01, *F*_(1, 16)_ = 0.1111, nonsignificant, respectively; main effect of inhibitor: *F*_(1, 16)_ = 0.6891, nonsignificant, *F*_(1, 16)_ = 0.3573, nonsignificant, respectively; interaction effects: *F*_(1, 16)_ = 0.01705, nonsignificant, *F*_(1, 16)_ = 0.1188, nonsignificant, respectively. **F** Main effect of agonist: *F*_(1, 16)_ = 0.08249, nonsignificant, *F*_(1, 16)_ = 60.22, *p* < 0.01, respectively; main effect of inhibitor: *F*_(1, 16)_ = 0.006845, nonsignificant, *F*_(1, 16)_ = 0.1733, nonsignificant, respectively; interaction effects: *F*_(1, 16)_ = 0.1695, nonsignificant, *F*_(1, 16)_ = 0.09182, nonsignificant, respectively. **G** Main effect of agonist: *F*_(1, 16)_ = 20.77, *p* < 0.01, *F*_(1, 16)_ = 15.69, *p* < 0.01, respectively; main effect of inhibitor: *F*_(1, 16)_ = 34.36, *p* < 0.01, *F*_(1, 16)_ = 7.981, *p* = 0.0122, respectively; interaction effects: *F*_(1, 16)_ = 22.98, *p* < 0.01, *F*_(1, 16)_ = 2.008, nonsignificant, respectively].

We first conducted the FST, which is known to induce a depression-like helplessness behavior (immobility) in naive mice, to assess the proper dose and administration time for KNT-127 under the present laboratory conditions. Compared to a single s.c. vehicle injection, single s.c. injection of KNT-127 (10 mg/kg) 30 min before the test (but not 24 h before the test) significantly decreased the immobility count (Fig. 1A), confirming an antidepressant-like effect. In contrast, this dose did not alter the locomotor activity of mice over 40 min post-injection (Fig. 1B). Moreover, the effect of KNT-127 on immobility in the FST was reversed by prior i.c.v. injection of the DOP inhibitor naltrindole (10 nmol/5 μL; Fig. 1C). Consistent with a previous study [5], these results indicate that KNT-127 exerts an antidepressant-like action through DOP stimulation.

### KNT-127 activates the mTOR signaling pathway in mPFC and amygdala

To determine whether the antidepressant-like action of KNT-127 requires mTOR-dependent pathway signaling, mice were injected i.c.v. with the selective mTOR inhibitor rapamycin 30 min before administration of s.c. KNT-127, and subsequently examined in the FST 30 min later. Rapamycin completely reversed KNT-127-mediated attenuation of the immobility count (Fig. 1D). Also consistent with a previous study [18], the antidepressant-like effect of ketamine observed 24 h after administration was also blocked by i.c.v. rapamycin (Supplementary Fig. S1). Subsequently, we analyzed the activation of mTOR signaling-related proteins in the mPFC, amygdala, and hippocampus (regions implicated in mood disorders) by western blotting. Subcutaneous injection of KNT-127 increased phosphorylation of Akt, a protein upstream of mTOR complex 1 (mTORC1), in the mPFC but not in the amygdala (Fig. 1E) and increased phosphorylation of ERK in the amygdala but not in the mPFC (Fig. 1F). Subcutaneous injection of KNT-127 also increased the phosphorylation of p70S6K, a protein kinase downstream of mTORC1, in both the mPFC and amygdala, and both responses were inhibited by i.c.v rapamycin (Fig. 1G). In contrast, these changes in phosphorylation were not observed in the hippocampus (Supplementary Fig. S2). Collectively, these findings strongly suggest the antidepressant-like effects of KNT-127 are mediated by Akt–mTORC1–p70S6K signaling in the mPFC and/or ERK–mTORC1–p70S6K signaling in the amygdala.

### Antidepressant-like effects of KNT-127 are inhibited by a phosphatidylinositol-3 kinase-Akt inhibitor

**Fig. 2:**
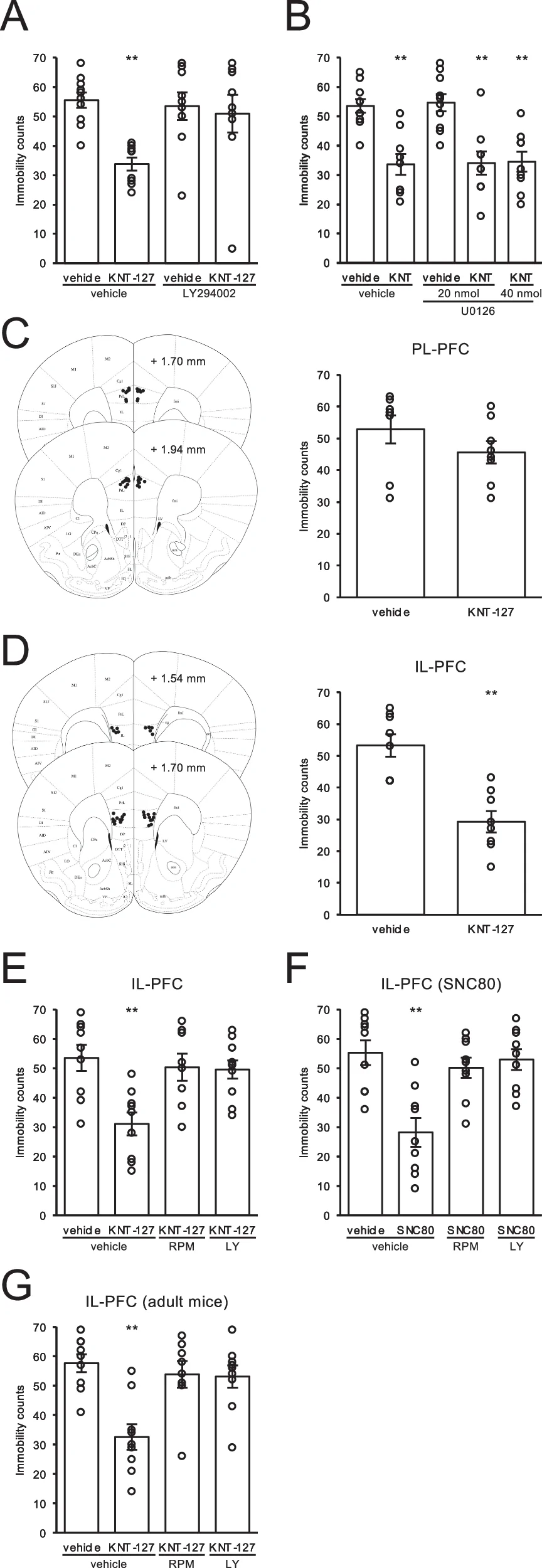
Antidepressant-like effects of delta opioid receptor (DOP) agonists in the forced swimming test (FST) are suppressed by blockade of phosphatidylinositol-3 kinase (PI3K) and mechanistic target of rapamycin (mTOR) signaling in infralimbic prefrontal cortex (IL-PFC). **A, B** Intracerebroventricular infusion of the PI3K inhibitor LY294002 (20 nmol; **A**), but not MEK inhibitor U0126 (20 or 40 nmol; **B**), 30 min before administration of KNT-127 (10 mg/kg, subcutaneously, 30 min before the test) blocked the antidepressant-like effects of KNT-127 in the FST. **C, D** Local infusion of KNT-127 (0.4 nmol, 15 min before the test) in the prelimbic prefrontal cortex (PL-PFC) **C** or IL-PFC **D**. Left panel: Cannula locations (injection sites) overlaid on images modified from the mouse brain atlas by Paxinos and Franklin [25]; right panel: immobility counts of mice in the FST. **E–G** Local infusion of the mTOR inhibitor rapamycin (RPM, 0.016 nmol, 20 min before the test) or LY294002 (LY, 1.6 nmol, 20 min before the test) into the IL-PFC reverses the antidepressant-like effects of KNT-127 (0.4 nmol, 15 min before the test; **E**) and the DOP agonist SNC80 (0.4 nmol, 15 min before the test; **F**). Comparison of effects on 6-week-old mice and 5-month-old mice **G**. Data for vehicle-pretreated groups were acquired from distinct experiments **A, B**, **E, F**. Data are presented as individual data points and the mean ± S.E.M. Statistical analyses were performed as follows: two-way factorial ANOVA followed by Dunnett’s *post-hoc* test compared with the vehicle-vehicle group **A, B**; Student’s *t*-test **C, D**; one-way factorial ANOVA followed by Dunnett’s *post-hoc* test compared with the vehicle-vehicle group **E–G**. ^**^p < 0.01. *n* = 8–10 mice per group. [(A) Main effect of agonist: *F*_(1, 33)_ = 8.147, *p* < 0.01; main effect of inhibitor: *F*_(1, 33)_ = 3.133, nonsignificant; interaction effect: *F*_(1, 33)_ = 5.078, *p* = 0.0310. **B** Main effect of agonist: *F*_(1, 34)_ = 40.03, *p* < 0.01; main effect of inhibitor: *F*_(1, 34)_ = 0.05808, nonsignificant; interaction effect: *F*_(1, 34)_ = 0.01046, nonsignificant. **E** *F*_(3, 32)_ = 6.535, *p* < 0.01. (F) *F*_(3, 32)_ = 9.406, *p* < 0.01. (G) *F*_(3, 31)_ = 8.482, *p* < 0.01].

To directly test whether these mTORC1 pathways are involved in the antidepressant-like actions of KNT-127, we measured the effects of Akt and ERK inhibition. Pretreatment with the phosphatidylinositol-3 kinase (PI3K) inhibitor LY294002 abolished the antidepressant-like effect of KNT-127 in the FST (Fig. 2A), whereas i.c.v. infusion of the MEK inhibitor U0126 did not (Fig. 2B). Therefore, the effects of KNT-127 are likely mediated, at least in part, through the PI3K–Akt pathway.

### Local infusion of KNT-127 in the IL-PFC produced antidepressant-like effects on behavior through PI3K and mTOR pathways

Based on these regional phosphorylation results, we tested whether KNT-127 exerts an antidepressant-like action when injected directly in the mPFC of cannula-implanted mice. A single microinfusion of KNT-127 into the PL-PFC did not significantly diminish the immobility count of mice (Fig. 2C); however, immobility was significant reduced by KNT-127 infusion into the IL-PFC (Fig. 2D), and this effect was inhibited by local infusion of rapamycin or LY294002 (Fig. 2E). Similar results were also obtained using another selective DOP agonist, SNC80 (Fig. 2F). Moreover, the PI3K- and mTOR-mediated antidepressant-like effects of KNT-127 were observed in 5-month-old adult mice (Fig. 2G), suggesting that KNT-127 exerts antidepressant-like effects through PI3K–mTORC1 signaling regardless of age.

### KNT-127 exerts antidepressant-like effects *via* the PI3K and mTOR pathway in an animal model of depression

**Fig. 3:**
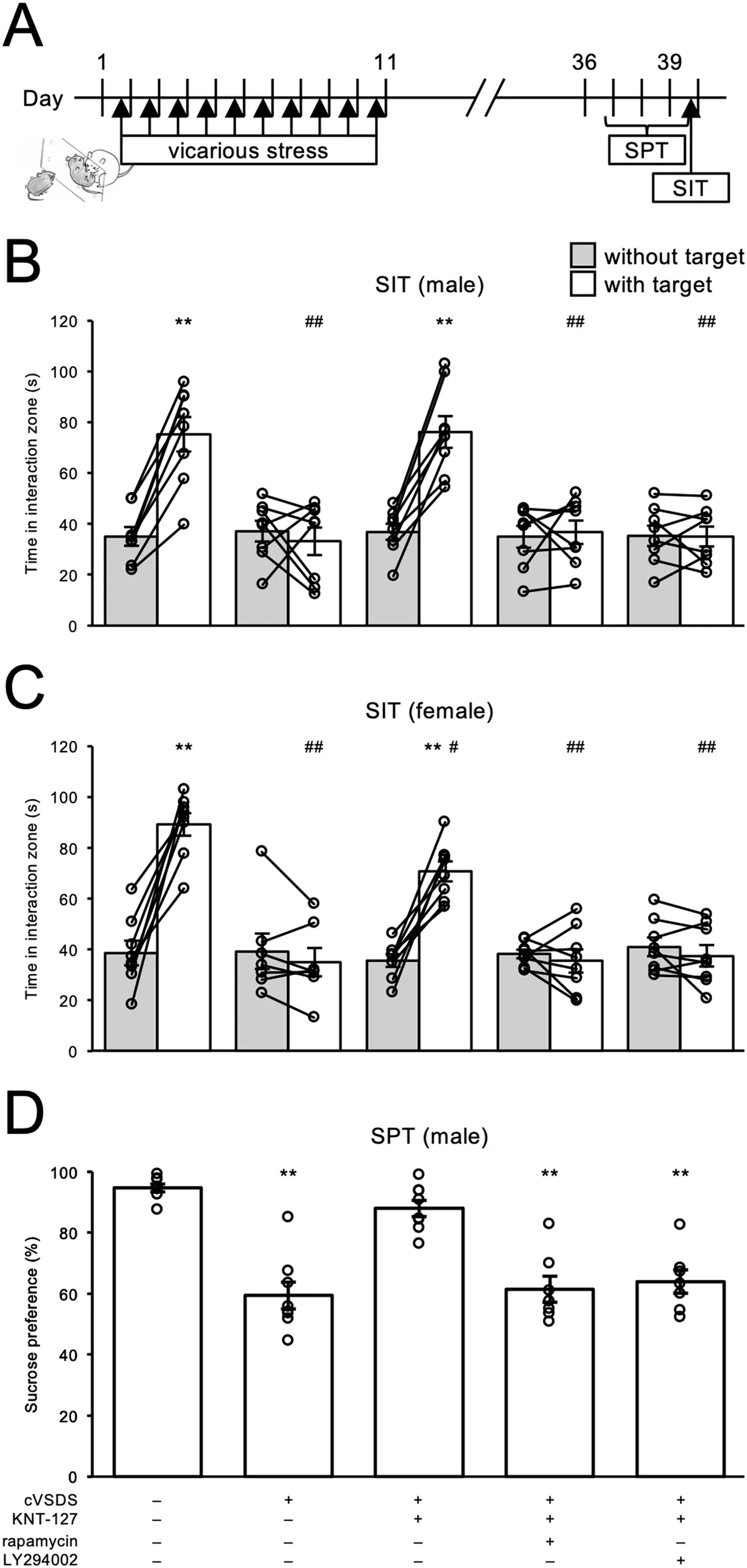
KNT-127 also exerts antidepressant-like effects in the chronic vicarious social defeat stress (cVSDS) mouse model of depression through phosphatidylinositol-3 kinase (PI3K) and mechanistic target of rapamycin (mTOR) signaling. **A** Schematic representation of the experimental design. SIT: social interaction test. SPT: sucrose preference test. **B**, **C** KNT-127 (10 mg/kg, subcutaneously, 30 min before the test) increases the time spent in the interaction zone in the presence of target mice in the SIT in both male **B** and female **C** cVSDS mice. **D** KNT-127 improves the cVSDS-induced attenuation of sucrose preference (anhedonia) in the SPT, and this response is also inhibited by rapamycin and LY294002. These effects were blocked by the mTOR inhibitor rapamycin [0.2 nmol, intracerebroventricularly (i.c.v.), 60 min before the test] and the PI3K inhibitor LY294002 (20 nmol, i.c.v., 60 min before the test). Data are presented as individual data points and the mean ± S.E.M. Statistical analyses were performed as follows: two-way repeated measures ANOVA followed by Bonferroni’s *post-hoc* test (*, no target vs. target; #, interaction time with social target compared with the naive-vehicle-vehicle group) **B, C**; one-way factorial ANOVA followed by Dunnett’s *post-hoc* test compared with the naive-vehicle-vehicle group **D**. ^#^*p* < 0.5 and ^**/##^*p* < 0.01. *n* = 7–8 mice per group. [**B** Main effect of the target: *F*_(1, 35)_ = 43.18, *p* < 0.01; main effect of the other conditions: *F*_(4, 35)_ = 8.055, *p* < 0.01; interaction effect: *F*_(4, 35)_ = 18.16, *p* < 0.01. **C** Main effect of the target: *F*_(1, 34)_ = 51.19, *p* < 0.01; main effect of the other conditions: *F*_(4, 34)_ = 10.82, *p* < 0.01; interaction effect: *F*_(4, 34)_ = 30.94, *p* < 0.01. **D** *F*_(3, 32)_ = 6.535, *p* < 0.01.].

We then examined if KNT-127 could reverse depression-like behaviors in an established depression model. A depression-like phenotype was established using the well-validated cVSDS protocol and subsequently tested using the SIT and SPT (Fig. 3A). KNT-127 increased the social interaction time with the target mouse in both male and female cVSDS mice, and this antidepressant-like response was again inhibited by i.c.v rapamycin or LY294002 (Fig. 3B, C). In addition, KNT-127 increased sucrose consumption in cVSDS mice, a behavioral sign of reduced depression-like anhedonia, and again this response was blocked by i.c.v. rapamycin or LY294002 (Fig. 3D). Therefore, the antidepressant-like effects of KNT-127 mediated by PI3K and mTOR signaling are not specific to strain, sex, or basal condition (stressed or unstressed).

### KNT-127 activates glutamatergic system in the IL-PFC *via* the mTOR signaling pathway and GABA_A_ receptors

**Fig. 4:**
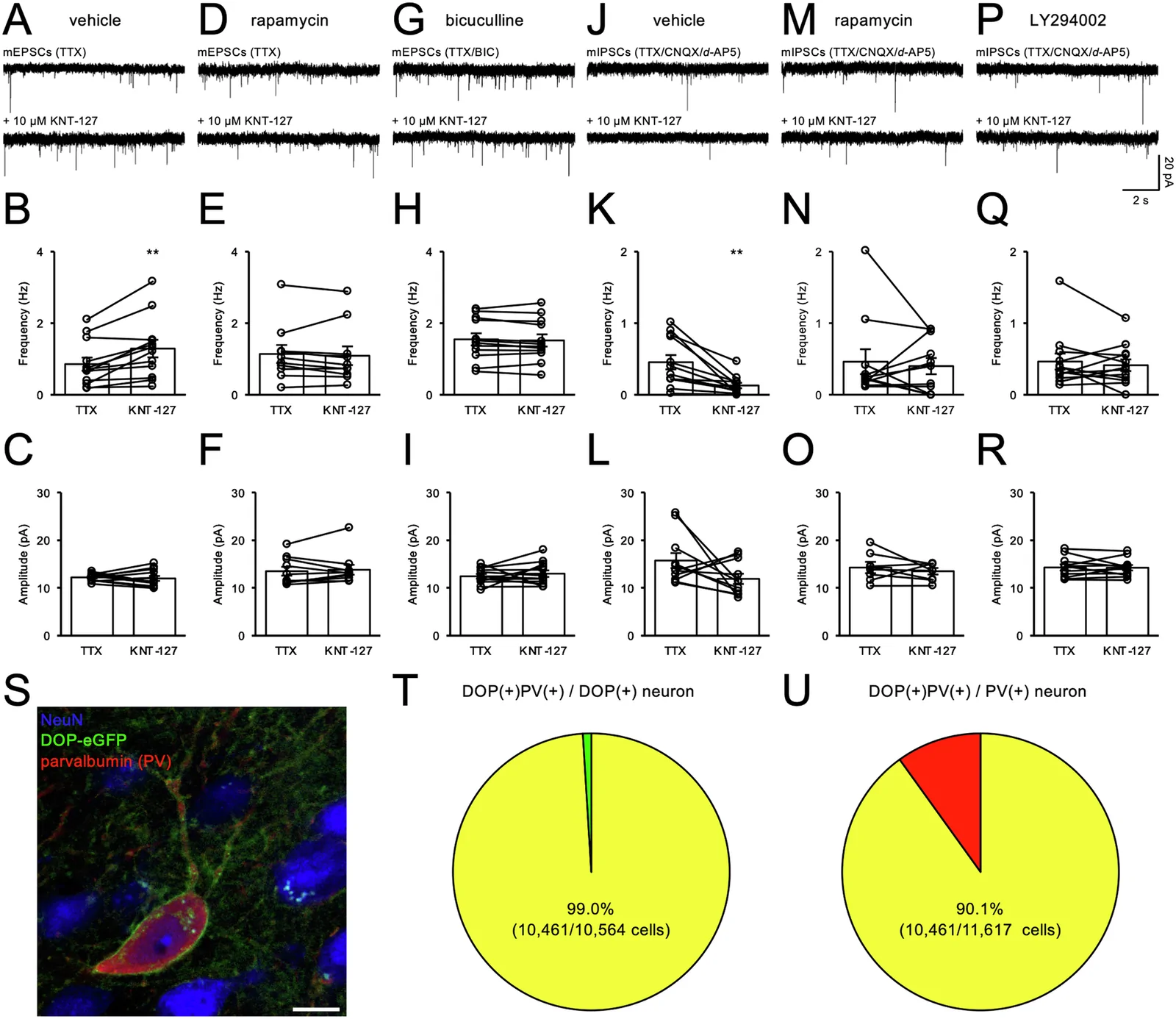
KNT-127 enhances glutamate release and suppresses gamma-aminobutyric acid (GABA) release in the infralimbic prefrontal cortex (IL-PFC) through activation of delta opioid receptors (DOPs) on parvalbumin (PV)-positive interneurons and ensuing phosphatidylinositol-3 kinase (PI3K) and mechanistic target of rapamycin (mTOR) signaling. **A–I** Miniature excitatory postsynaptic currents (mEPSCs) in the vehicle-pretreated group [5% DMSO, intracerebroventricularly (i.c.v.), 30 min before the sacrifice; **A–C**], the mTOR inhibitor (rapamycin)-pretreated group (0.2 nmol, i.c.v., 30 min before the sacrifice; **D–F**), and the GABA_A_ receptor inhibitor (bicuculline)-perfused group (5% DMSO, i.c.v., 30 min before the sacrifice; 50 μM bath application; **G–I**). **J–R** Miniature inhibitory postsynaptic currents (mIPSCs) in the vehicle-pretreated group (5% DMSO, i.c.v., 30 min before the sacrifice; **J–L**), the rapamycin-pretreated group (0.2 nmol, i.c.v., 30 min before the sacrifice; **M–O**), and PI3K inhibitor (LY294002)-pretreated group (20 nmol, i.c.v., 30 min before the sacrifice; **P–R**). **A, D, G, J, M, P** Representative traces. **B, E, H, K, N, Q** Mean mEPSC or mIPSC frequency. **C, F, I, L, O, R** Mean mEPSC or mIPSC amplitude. BIC: bicuculline, TTX: tetrodotoxin. Data are presented as individual data points and the mean ± S.E.M. Statistical analyses were performed using a paired sample *t*-test. ^**^p < 0.01. *n* = 10–12 cells from 6–8 mice. **S** A representative image of DOP/PV double-positive neurons. Scale bar: 10 μm. **T, U** Proportions (%) of DOP/PV double-positive neurons to DOP-positive neurons **T** or PV-positive neurons **U** in the IL-PFC. *n* = 11,720 cells from 6 mice (male: *n* = 3, female: *n* = 3).

To determine the physiological mechanism underlying the antidepressant-like actions of KNT-127, we evaluated the effects on mEPSCs and mIPSCs in pyramidal neurons in layer II/III or layer V of the IL-PFC. The mean frequency of mEPSCs was significantly and dose-dependently increased by perfusion of IL-PFC slices with KNT-127, while amplitude, rise time, and decay time were unaltered (Fig. 4A–C; Supplementary Figs. S3 and S4A, B). This result suggests that KNT-127 promotes glutamatergic system activity without altering the gating properties of postsynaptic glutamate receptors. Furthermore, pretreatment with rapamycin (i.c.v.) or bath perfusion of the GABA_A_ receptor blocker bicuculline reversed the increase in mEPSC frequency without affecting amplitude, rise time, or decay time (Fig. 4D–I; Supplementary Fig. S4C–F), suggesting that the enhanced glutamatergic transmission onto IL-PFC pyramidal neurons results from reduced local GABAergic transmission.

### KNT-127 suppresses GABAergic synapse currents in the IL-PFC

We then examined if this KNT-127-induced increase in IL-PFC pyramidal neuron excitability is dependent on GABAergic suppression by measuring mIPSCs in the presence of glutamate receptor antagonists. Indeed, the mean frequency of mIPSCs (but not amplitude and kinetics) was significantly reduced by perfusion of KNT-127 (Fig. 4J–L; Supplementary Fig. S4G, H), and this effect was inhibited by pretreatment with rapamycin or LY294002 (Fig. 4M–R; Supplementary Fig. S4I–L). These results suggest that KNT-127 suppresses the release of GABA through PI3K and mTOR signaling, leading to enhances glutamatergic transmission onto IL-PFC pyramidal neurons.

### DOPs are mainly expressed by PV-positive interneurons in the IL-PFC

Finally, to identify the cellular site of these effects within the IL-PFC, we investigated the distribution of DOPs in the IL-PFC of transgenic mice with DOP-promoter-driven enhanced green fluorescence protein expression (DOP-eGFP mice) by immunostaining for the interneuron marker PV (Fig. 4S). Approximately 99% of eGFP-positive neurons were PV-positive (10,461/10,564 cells; Fig. 4T), and vice versa 90% of PV-positive neurons were eGFP-positive (10,461/11,617 cells; Fig. 4U), suggesting that DOPs are primarily expressed by PV-expressing GABAergic interneurons in the IL-PFC.

## Discussion

We and others have proposed that the DOP is a promising therapeutic target for mood disorders such as depression due to rapid onset and potentially fewer adverse effects than benzodiazepine anxiolytics and monoaminergic antidepressants. In the present study, we elucidated the functional mechanisms underlying the antidepressant-like activity of selective DOP agonists. Systemic administration of KNT-127 attenuated behavioral helplessness (immobility) in the FST, and this response was inhibited by prior i.c.v. treatment with the mTOR inhibitor rapamycin. In addition, systemic KNT-127 increased phosphorylation levels of the mTORC1-associated signaling factors Akt and p70S6K in the mPFC, and these responses were also inhibited by i.c.v. rapamycin. The PI3K inhibitor LY294002 also blocked the antidepressant-like effects of KNT-127, suggesting that the DOP–PI3K–Akt–mTORC1–p70S6K signaling cascade is essential for this activity. Further, mTOR and PI3K inhibition also reduced the antidepressant-like effects of KNT-127 in an animal model of depression as assessed by SIT and SPT. Local infusion of either KNT-127 or SNC80 into the IL-PFC exerted robust antidepressant-like effects in the FST that again were reversed by rapamycin and LY294002. Direct administration of KNT-127 to isolated IL-PFC tissue accelerated glutamatergic transmission and suppressed GABAergic transmission to cortical pyramidal neurons, and these effects were similarly blocked by PI3K and mTOR inhibitors. Finally, we found that DOPs were mainly expressed by PV-positive interneurons in the IL-PFC, implying that reduced presynaptic GABA release due to DOP–PI3K–Akt–mTORC1–p70S6K signal activation is the primary mechanism underlying the excitatory shift observed in pyramidal neurons.

The IL-PFC in rodents is considered the functional equivalent of Brodmann Area 25 in humans based on documented functions in mood regulation and common projections to other brain regions [34, 35]. Clinical studies have reported that Brodmann Area 25 correlates with mood impairment and treatment resistance in patients with MDD [36, 37]. A structural and functional neuroimaging study further reported that this area is atrophied and hypoactive in patients with MDD [38]. The antidepressant effects of ketamine were also accompanied by enhanced synaptic responses of pyramidal neurons in the rat IL-PFC [39], while single bilateral infusion of *R*-ketamine into the IL-PFC improved learned helplessness-like symptoms in rats [40]. In the present study, we revealed that DOPs in the IL-PFC play an important role in production of antidepressant-like effects.

Numerous studies have proposed that depression is associated with an excitatory–inhibitory neurotransmission imbalance within the mPFC [41]. We found that DOP activation enhanced the frequency of mEPSCs while suppressing mIPSC frequency as measured in IL-PFC pyramidal neurons without altering response amplitude or kinetics, suggesting that these effects are presynaptic. Moreover, these responses were completely blocked by mTOR inhibition, so it is reasonable to conclude that the DOPs and downstream signaling cascades mediating these effects are presynaptic to pyramidal neurons. In addition, KNT-127 exhibited similar actions in both layer II/III and layer V of the IL-PFC (Supplementary Fig. S5). We further speculate that these effects are mediated by PV-positive interneurons. First, PV-positive interneurons account for a large proportion of all interneurons in the IL-PFC, are distributed equally in layer II/III and layer V [42, 43], and are known to maintain the excitatory–inhibitory balance of pyramidal neurons [44, 45]. Also, chronic stress was previously reported to induce hyperactivation of these cells, resulting in IL-PFC hypofunction and depression [45–48]. We confirmed a major contribution of PV-positive interneurons to the overall antidepressant-like effects of DOP activation by showing that the majority of PV-positive interneurons in the IL-PFC expressed DOPs and that the vast majority of DOP-positive interneurons expressed PV. To our best knowledge, this is the first study to report cell-specific expression of DOPs in a single brain region. These results identify DOP- and PV-expressing interneurons as critical therapeutic targets for mood disorders. To validate our hypothesis, PV-positive neuron-specific DOP or mTOR (or Raptor) knockdown experiments will be needed to eliminate other possibilities, such as DOP-positive/PV-negative neurons or postsynaptic mTORC1 signaling roles. Recently, Nawreen et al. reported that acute chemogenetic inhibition of PV-positive interneurons in the IL-PFC increased passive coping behaviors in the mice tail suspension test, whereas chronic inhibition during the chronic variable stress period decreased immobility in the FST [49]. This finding in the acute paradigm is partly inconsistent with our results, possibly due to variations in experimental conditions. At the same time, we previously reported that repeated administration of KNT-127 during the cVSDS period produces anti-stress effects in the social interaction test [31]. Altogether, the primary conclusion drawn from both the current study and the preceding report consistently emphasizes the crucial role of PV-positive interneurons in the IL-PFC in regulating the excitatory-inhibitory balance which is implicated in stress-induced depressive-like states.

The serine/threonine kinase mTOR is a component of at least two signaling complexes, mTORC1 and mTORC2. McCabe and colleagues reported that genetic inactivation of either mTORC1 or mTORC2 in cultured glutamatergic hippocampal neurons reduced evoked EPSCs but that this response was mediated by distinct mechanisms; the effects of mTORC1 were postsynaptic and mTORC2 were presynaptic [50]. On the other hand, the presynaptic protein synthesis required for long-term depression of GABAergic signaling was found to be regulated by the mTORC1 pathway, not the mTORC2 pathway [51, 52]. In the present study, we demonstrated that presynaptic mTOR is involved in the antidepressant-like effects of DOP agonists. Akt is implicated in two mTOR-related pathways, PI3K–Akt–mTORC1 and mTORC2–Akt–mTORC1, while p70S6K is a downstream target of mTORC1 [53]. In our study, both the PI3K and mTOR inhibitor blocked the antidepressant-like actions of DOP agonists. Given that an acute administration of rapamycin specifically inhibits mTORC1 [52, 54], the antidepressant-like effects of DOP agonists were mediated by downstream activation of the PI3K–Akt–mTORC1–p70S6K pathway in the IL-PFC. KNT-127 also ameliorated social interaction deficits and anhedonia-like symptoms through the same pathway in cVSDS mice, a widely accepted and validated animal model of depression with strong constructive, face, and predictive validity [55]. Moreover, KNT-127 produced similar antidepressant-like effects in adult as well as juvenile mice and both male and female mice. Therefore, these findings support the robustness of DOP activation as an antidepressant strategy and provide highly plausible underlying mechanisms that define additional therapeutic targets.

**Fig. 5:**
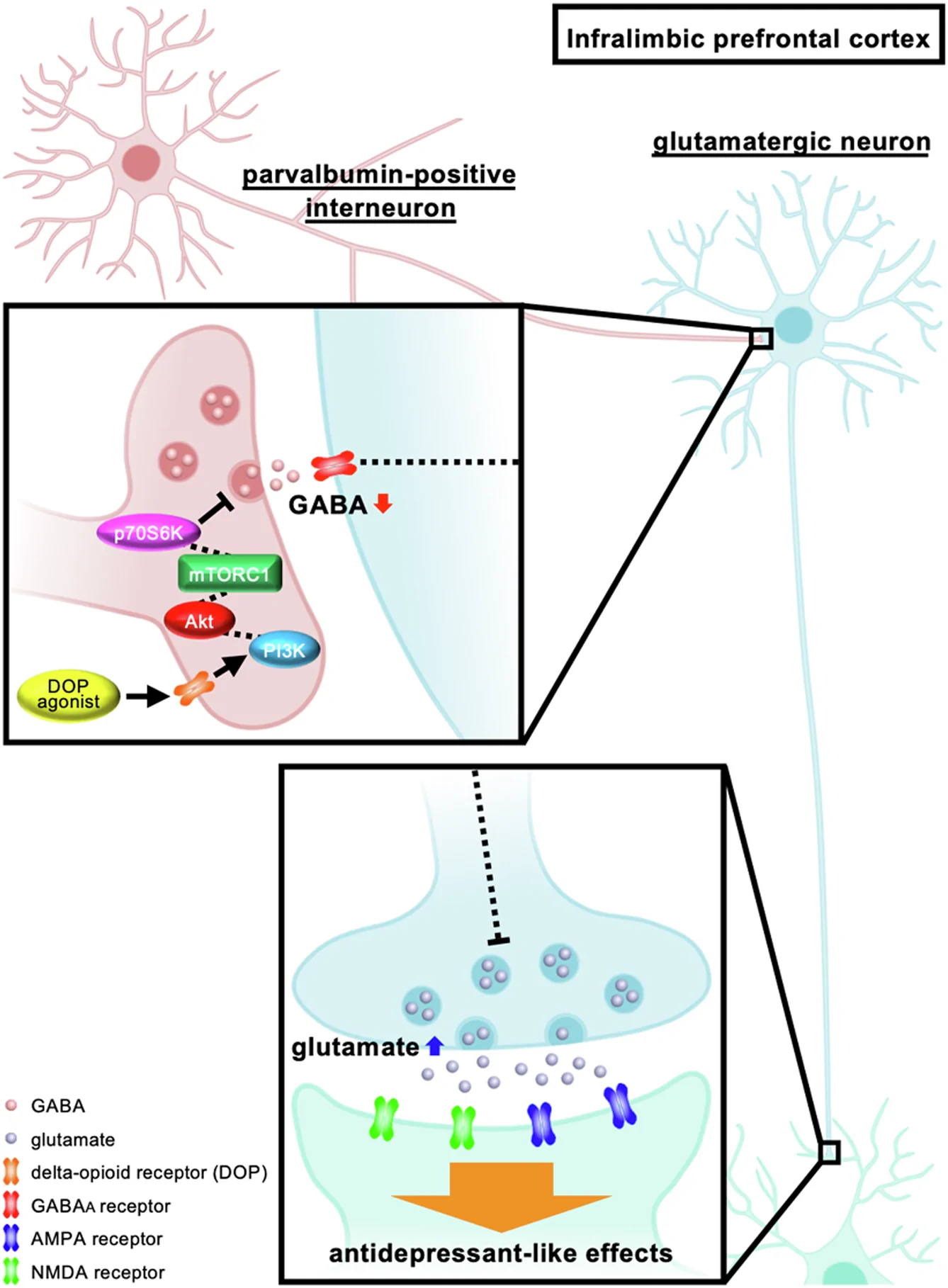
Proposed mechanism underlying the antidepressant-like effects of delta opioid receptor (DOP) agonists. Activation of DOPs attenuates gamma-aminobutyric acid (GABA) release from parvalbumin-positive interneurons in the infralimbic prefrontal cortex through activation of a signaling pathway involving phosphatidylinositol-3 kinase (PI3K), Akt, mechanistic target of rapamycin complex 1 (mTORC1), and p70S6 kinase (p70S6K). Consequently, glutamate release is relatively accelerated, leading to greater pyramidal neuron excitability and antidepressant-like effects on mouse behavior.

Based on our findings, we proposed the following hypothesis. The antidepressant-like effects of DOP agonists involve the activation of DOPs in PV-positive interneurons of the IL-PFC, which activates the PI3K–Akt–mTORC1–p70S6K signaling pathway and reduces presynaptic release of GABA. This in turn facilitates glutamatergic transmission onto pyramidal neurons (Fig. 5). The antidepressant ketamine has also been reported to activate mTOR signaling in the IL-PFC and hippocampus; however, ketamine activates mTOR at postsynaptic sites of glutamatergic neurons in the mPFC, a mechanism that contributes to the rapid and sustained (hours to seven days) but not acute (within 1 h) effects of this agent [18, 19, 56]. Besides, ketamine activates both Akt and ERK signaling in the mPFC [18], whereas KNT-127 increases the phosphorylation levels of Akt, but not ERK. Furthermore, eukaryotic initiation factor 4E-binding proteins acting downstream mTORC1 are implicated in the acute and sustained effects of ketamine on excitatory and inhibitory neurons in the hippocampus associated with antidepressant activity [57], while we found that KNT-127 did not influence mTOR signaling in the hippocampus. We also reported that neither single nor repeated administration of KNT-127 enhances adult hippocampal neurogenesis in either naive or cVSDS mice, in contrast to ketamine [19, 31]. Thus, while mTOR signaling is implicated in the antidepressant-like effects of both DOP agonists and ketamine, the cellular action sites are distinct, which may explain differences in potency, therapeutic duration, and side effects. However, it is premature to draw conclusions regarding differences in onset times and durations between KNT-127 and ketamine without more extensive studies of acute versus chronic effects on the same experimental models. Further, it is premature to conclude that there are no shared mechanisms underlying antidepressant-like activities.

The seminal finding of this study is that DOPs facilitate neuronal excitability in the IL-PFC by activating the PI3K–mTORC1 pathway in local GABAergic interneurons, resulting in acute antidepressant-like behavioral effects. These findings do not preclude additional action mechanisms, although our local infusion experiments demonstrate that this IL-PFC pathway is essential for the behavioral antidepressant-like effects of DOP activation. Further studies are required to identify other regions and pathways contributing to the antidepressant-like actions of selective DOP agonists such as KNT-127.

We found that the MEK-ERK-mTORC1-p70S6K pathway in the amygdala is not involved in the antidepressant-like effects of KNT-127. In contrast, we previously reported that KNT-127 exerts robust anxiolytic-like effects [10, 12] and facilitates the extinction of contextual fear memory through ERK signaling in the amygdala [27, 58]. We also found that KNT-127 suppresses glutamate release in the PL-PFC, although it is unclear if this response also involves mTOR [12, 59]. The PL-PFC–amygdala circuit modulates anxiety-like behavior [60], so we speculate that activation of the amygdala ERK–mTORC1–p70S6K pathway contributes to the anxiolytic-like effects of DOP agonists. Indeed, Ko and coworkers demonstrated that ERK activation in the amygdala is critical for DOP-induced anxiolytic-like effects [61]. We also previously proposed that the mechanisms underlying the antidepressant-like and anxiolytic effects of DOP agonists may be different; for example, the PI3K–Akt signaling in the IL-PFC is not implicated in the anxiolytic-like effects of KNT-127 [58]. Therefore, further studies are required to clarify the region-specific responses to DOP agonists and the ensuing effects on mood. Furthermore, such studies should address one limitation of our study, the use of anesthetized mice for i.c.v. injections as anesthetics may influence cellular signaling processes such as MAPK phosphorylation.

In conclusion, we present evidence that DOP agonists exert antidepressant-like effects through activation of the PI3K–Akt–mTORC1–p70S6K signaling cascade in PV-positive GABAergic interneurons of the IL-PFC, reducing GABAergic transmission and enhancing the excitability of local pyramidal neurons. These results provide a foundation for more detailed elucidation of the antidepressant mechanisms of DOPs and the development of novel DOP-targeted antidepressants with improved side effects profiles for MDD.

## Supplementary information

Supplemental Information

Supplementary Figure S1

Supplementary Figure S2

Supplementary Figure S3

Supplementary Figure S4

Supplementary Figure S5

## Data Availability

All data generated during and analyzed in the current study are available from the corresponding author upon reasonable request.
