# Correction: Delta opioid receptor agonists activate PI3K–mTORC1 signaling in parvalbumin-positive interneurons in mouse infralimbic prefrontal cortex to exert acute antidepressant-like effects

**DOI:** 10.1038/s41380-025-03046-5

**Published:** 2025-05-08

**Authors:** Toshinori Yoshioka, Daisuke Yamada, Akari Hagiwara, Keita Kajino, Keita Iio, Tsuyoshi Saitoh, Hiroshi Nagase, Akiyoshi Saitoh

**Affiliations:** 1 Laboratory of Pharmacology, Faculty of Pharmaceutical Sciences, Tokyo University of Science, Chiba, Japan; 2 Department of Applied Biological Science, Faculty of Science and Technology, Tokyo University of Science, Chiba, Japan; 3 International Institute for Integrative Sleep Medicine (WPI-IIIS), University of Tsukuba, Ibaraki, Japan

**Keywords:** Neuroscience, Molecular biology

Correction to: *Molecular Psychiatry* (2024) **30**:2038-2048 10.1038/s41380-024-02814-z, published online 06 December 2024

In this article the title was incorrectly given as ‘Delta opioid receptor agonists activate PI3K–mTORC1 signaling in parvalbumin-positive interneurons in mouse infralimbic prefrontal cortex to exert acute antidepressant-lie effects’ but should have been ‘Delta opioid receptor agonists activate PI3K–mTORC1 signaling in parvalbumin-positive interneurons in mouse infralimbic prefrontal cortex to exert acute antidepressant-like effects’. The original article has been corrected.

