# Supplementary material for: Delta opioid receptor agonists activate PI3K–mTORC1 signaling in parvalbumin-positive interneurons in mouse infralimbic prefrontal cortex to exert acute antidepressant-like effects
Source: Mol Psychiatry. 2024 Dec 6;30(5):2038–48. doi: 10.1038/s41380-024-02814-z (PMC12015109; doi:10.1038/s41380-024-02814-z)
Supplement: Supplementary file 1 — Supplemental Information [file 41380_2024_2814_MOESM1_ESM.docx]

**Supplemental Information**

**Materials and methods**

***Forced swimming test (FST)***—A 5-L glass cylinder (diameter, 175 mm; height, 265 mm) filled with 25°C ± 1°C water to a depth of 131 mm (3 L) was used as an inescapable swimming apparatus. On the first day, ICR mice were placed individually in the apparatus for 15 min as a training session. Twenty-four hours later, each animal was placed into the apparatus for 10 min as a test session, and dominant behaviors were counted manually at 5 s intervals during the final 6 min. Immobility was defined as the cessation of all active behavior and floating with minimal movement.

***Locomotor activity measurement***—Naive ICR mice were placed individually in a clean acrylic cage (310 × 205 × 125 mm) and acclimatized for 2 h. Following drug administration, locomotion was assessed every 5 min for 40 min using an automatic tracking system (Muromachi Kikai, Tokyo, Japan).

***Chronic vicarious social defeat stress paradigm and behavioral tests***—To establish the cVSDS model, a C57BL/6J mouse was placed on one side of the home cage containing an aggressive ICR mouse with the mice separated by a perforated acryl divider, and then another substitute C57BL/6J mouse was placed in with the ICR mouse. This procedure was performed for 10 min and repeated for 10 consecutive days. For the SIT, a mouse was placed in an interaction field (450 × 450 mm) with wire-mesh cage at one end. The movements of the mouse were tracked for 2.5 min before an unfamiliar ICR target mouse was placed in the wire-mesh cage, after which the movements were tracked for another 2.5 min. The time spent in the 140 × 240 mm zone around the wire-mesh cage (the interaction zone) was measured using SCANET MV-40 (Melquest Ltd., Toyama, Japan).

***Acute slice preparation and whole-cell patch-clamp recordings***—Mice were deeply anesthetized with isoflurane, and brains rapidly extracted. Freshly excised brains were placed in cold artificial cerebrospinal fluid (aCSF: 124 mM NaCl, 3 mM KCl, 1.2 mM KH2PO4, 26 mM NaHCO3, 2 mM CaCl2, 1.3 mM MgSO4, and 10 mM glucose; pH 7.4; 280–310 mosmol/L) continuously aerated with 95% O2/5% CO2 for 2 min. Subsequently, a 300-μm thick coronal section containing the IL-PFC was obtained using a vibrating blade tissue slicer (LinearSlicer PRO7N, DOSAKA EM, Kyoto, Japan) and maintained in aCSF for at least 30 min at room temperature as a recovery period. The slice was placed in a recording chamber and perfused with aCSF maintained at 32°C and flowing at 2–4 mL/min by gravity feed. Pyramidal neurons in layer II/III or layer V of the IL-PFC were identified by infrared/differential interference contrast (IR/DIC) microscopy (×400; BX51WI, Olympus, Tokyo, Japan) and patch-clamped with live visual guidance. Pipette solutions for intracellular dialysis and recording protocols are detailed in the next two sections. All output signals were amplified and filtered at 2 kHz using an AXOPATCH 200B patch-clamp amplifier (Molecular Devices, San Jose, CA, USA), digitized at 5 kHz, and recorded using Clampex software version 9.2 (Molecular Devices). Series resistance was monitored frequently during recording and was usually 11–20 MΩ. Cells were discarded from the analysis if series resistance changed by >20% or exceeded 25 MΩ.

***Immunohistochemistry***—DOP-eGFP mice were anesthetized by intraperitoneal injection of medetomidine (0.75 mg/kg), midazolam (4 mg/kg), and butorphanol (5 mg/kg), then transcardially perfused with 9.25% sucrose solution followed by 4% paraformaldehyde in 0.1 M phosphate buffer (pH 7.4). Brains were excised and cryosectioned at 30-μm thickness in the coronal plane through the entire IL-PFC (AP: +1.98 mm to +1.34 mm from the bregma26; 21 sections) with a cryostat (CM3050 S, Leica Biosystems, Wetzlar, Germany). Thereafter, all sections were blocked with 5% normal donkey serum in PBS containing 0.1% Triton-X100 for 1 h at room temperature, incubated with mouse monoclonal anti-NeuN (1:1000; MAB377, Merck KGaA) and guinea pig polyclonal anti-parvalbumin (PV; 1:1000; 010-28561, FUJIFILM Wako Pure Chemical) at 4°C overnight, and then with secondary antibody conjugated to AlexaFluor 405 (anti-mouse; 1:1000; A31553, Thermo Fisher Scientific) or AlexaFluor 564 (anti-guinea pig; 1:1000; A11074, Thermo Fisher Scientific) for 1 h at room temperature.
