# Supplementary figures and images for: Delta opioid receptor agonists activate PI3K–mTORC1 signaling in parvalbumin-positive interneurons in mouse infralimbic prefrontal cortex to exert acute antidepressant-like effects
Source: Mol Psychiatry. 2024 Dec 6;30(5):2038–48. doi: 10.1038/s41380-024-02814-z (PMC12015109; doi:10.1038/s41380-024-02814-z)

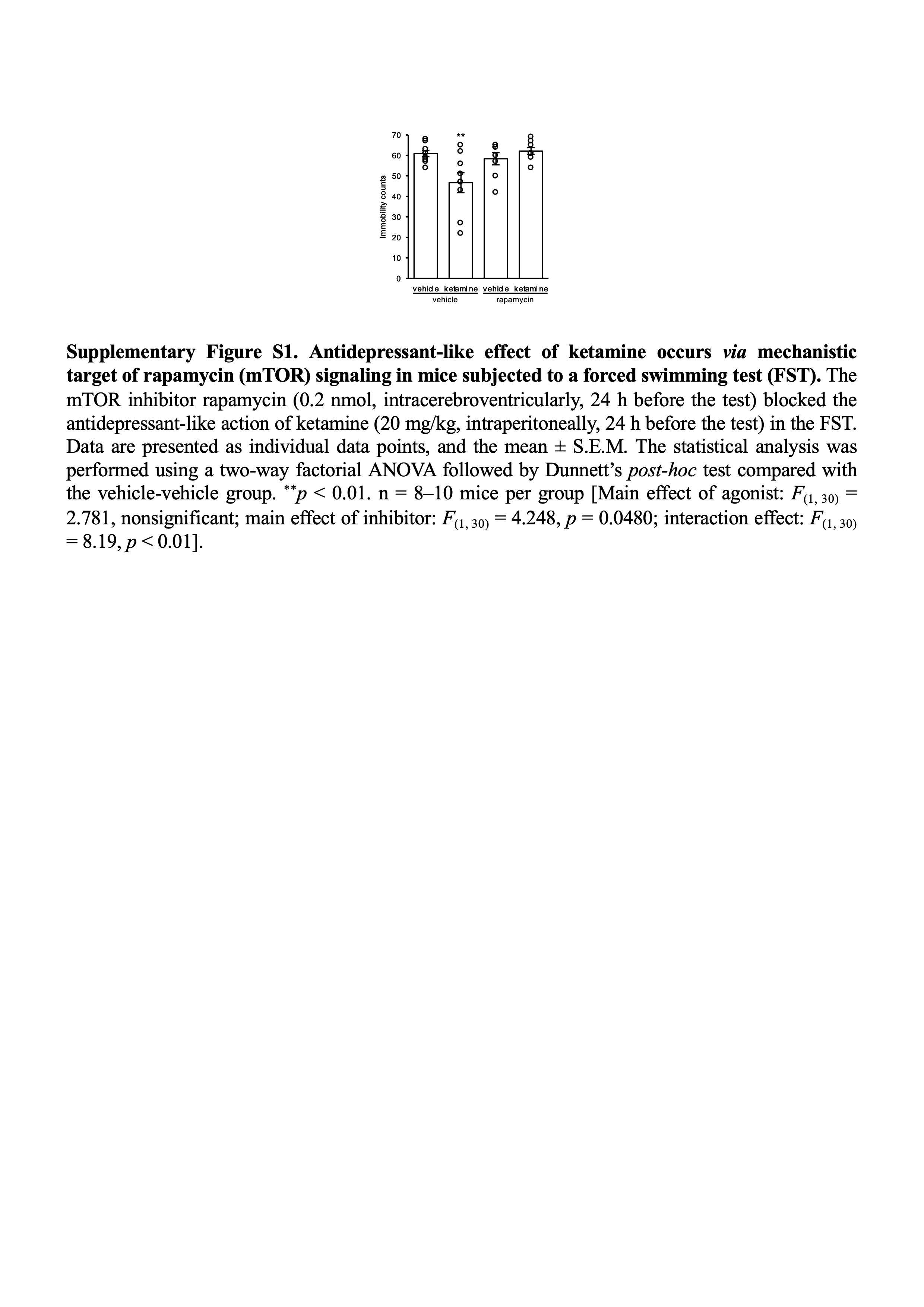

Supplement: Supplementary file 2 — Supplementary Figure S1 [file 41380_2024_2814_MOESM2_ESM.tif]

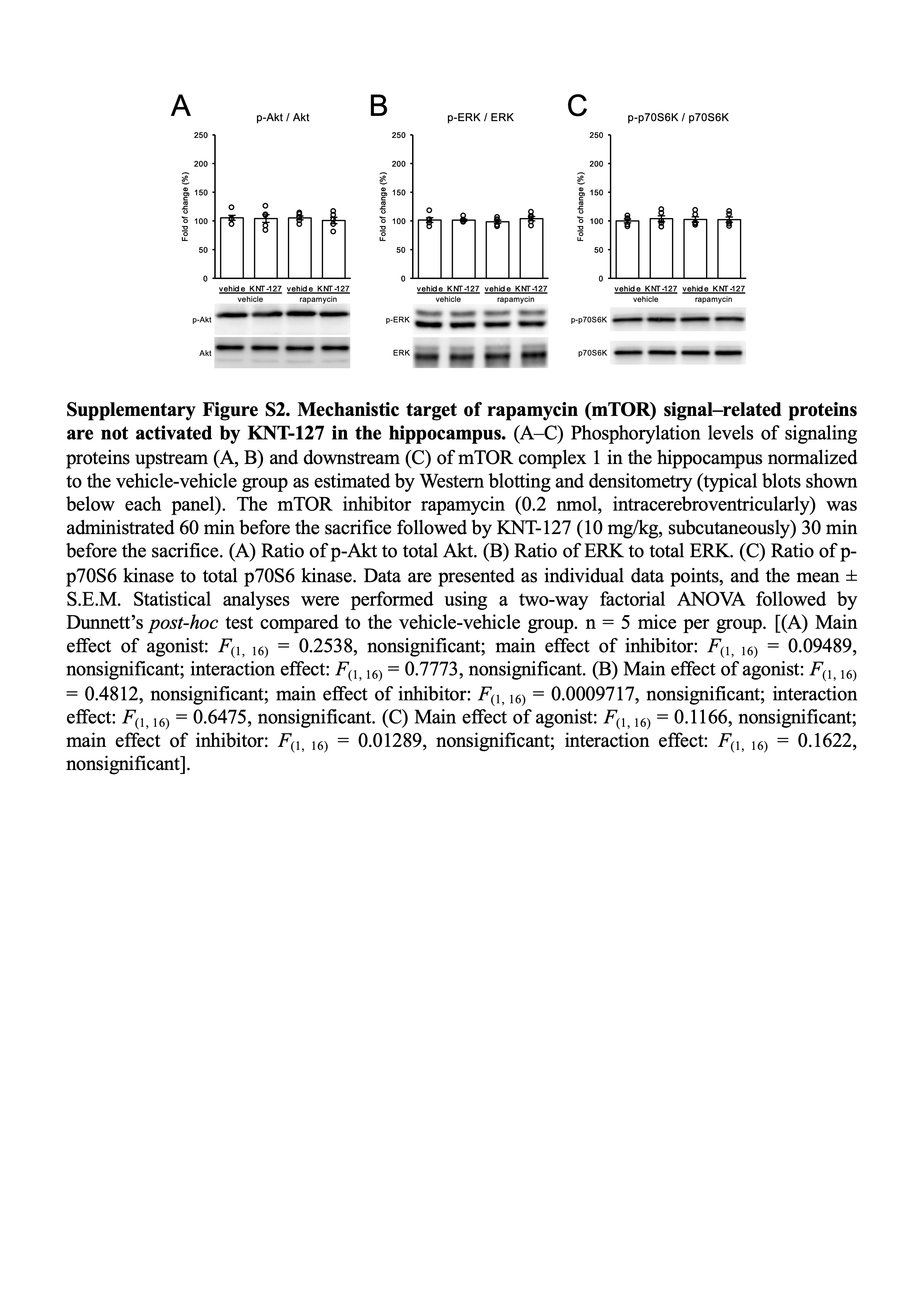

Supplement: Supplementary file 3 — Supplementary Figure S2 [file 41380_2024_2814_MOESM3_ESM.tif]

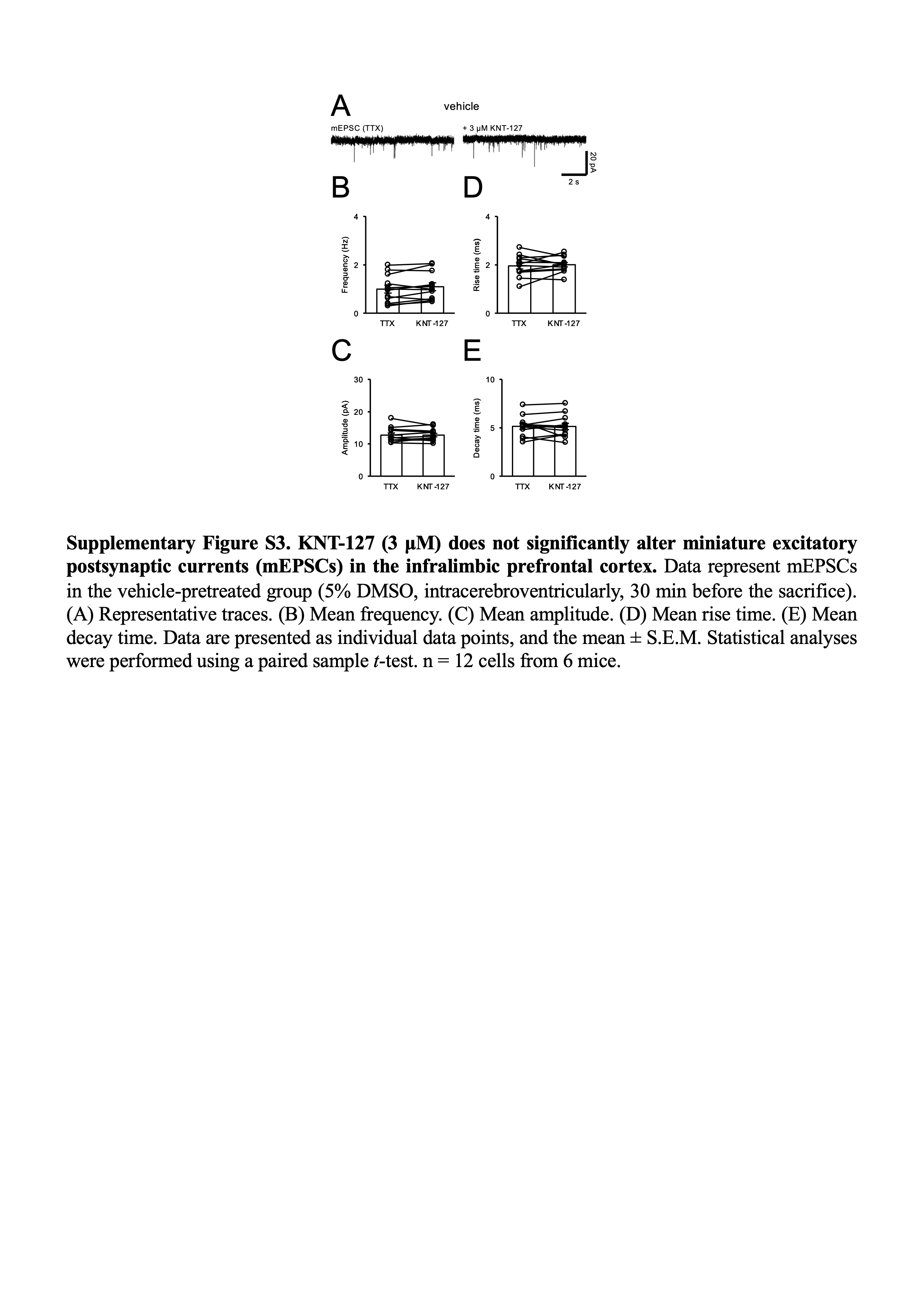

Supplement: Supplementary file 4 — Supplementary Figure S3 [file 41380_2024_2814_MOESM4_ESM.tif]

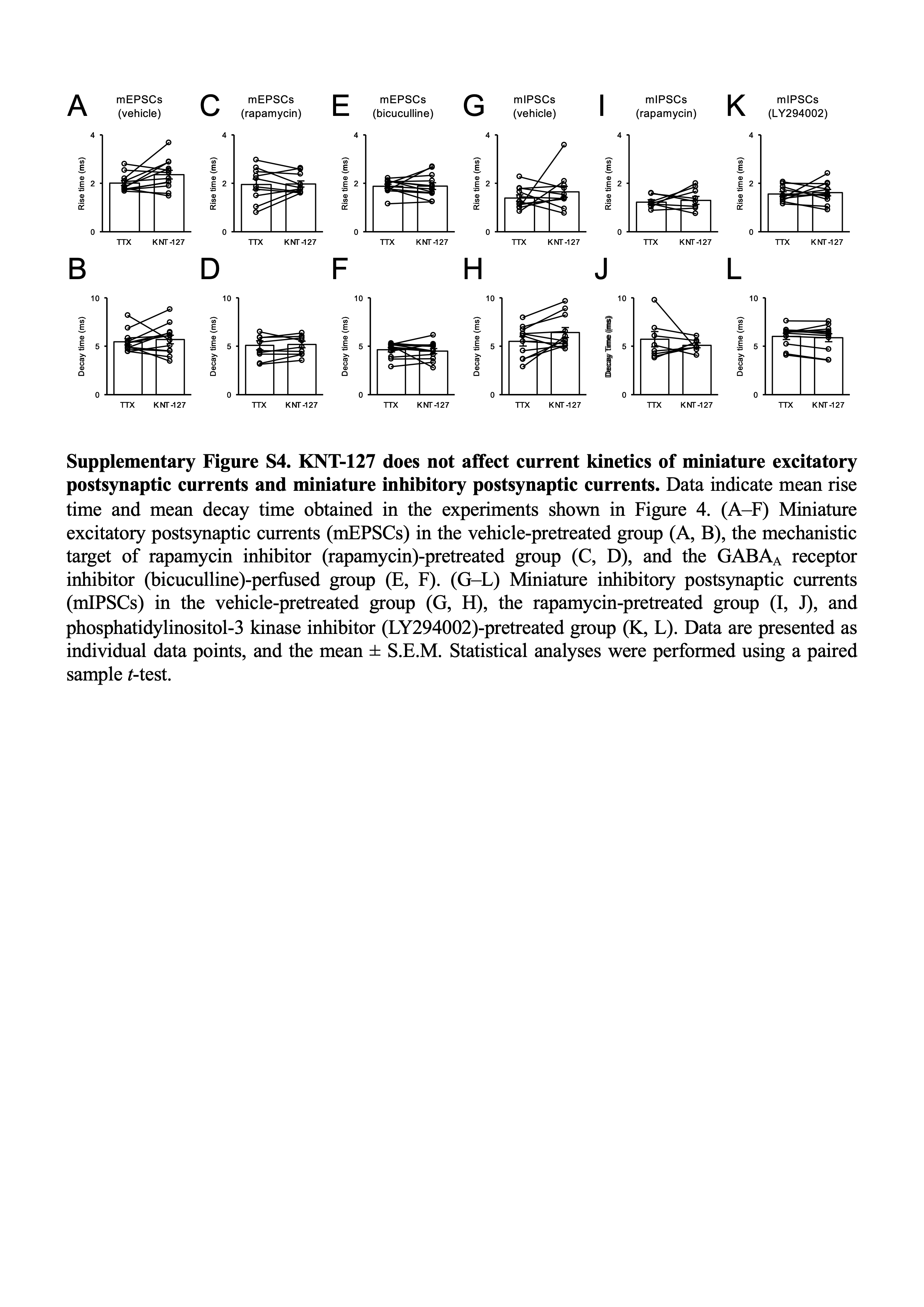

Supplement: Supplementary file 5 — Supplementary Figure S4 [file 41380_2024_2814_MOESM5_ESM.tif]

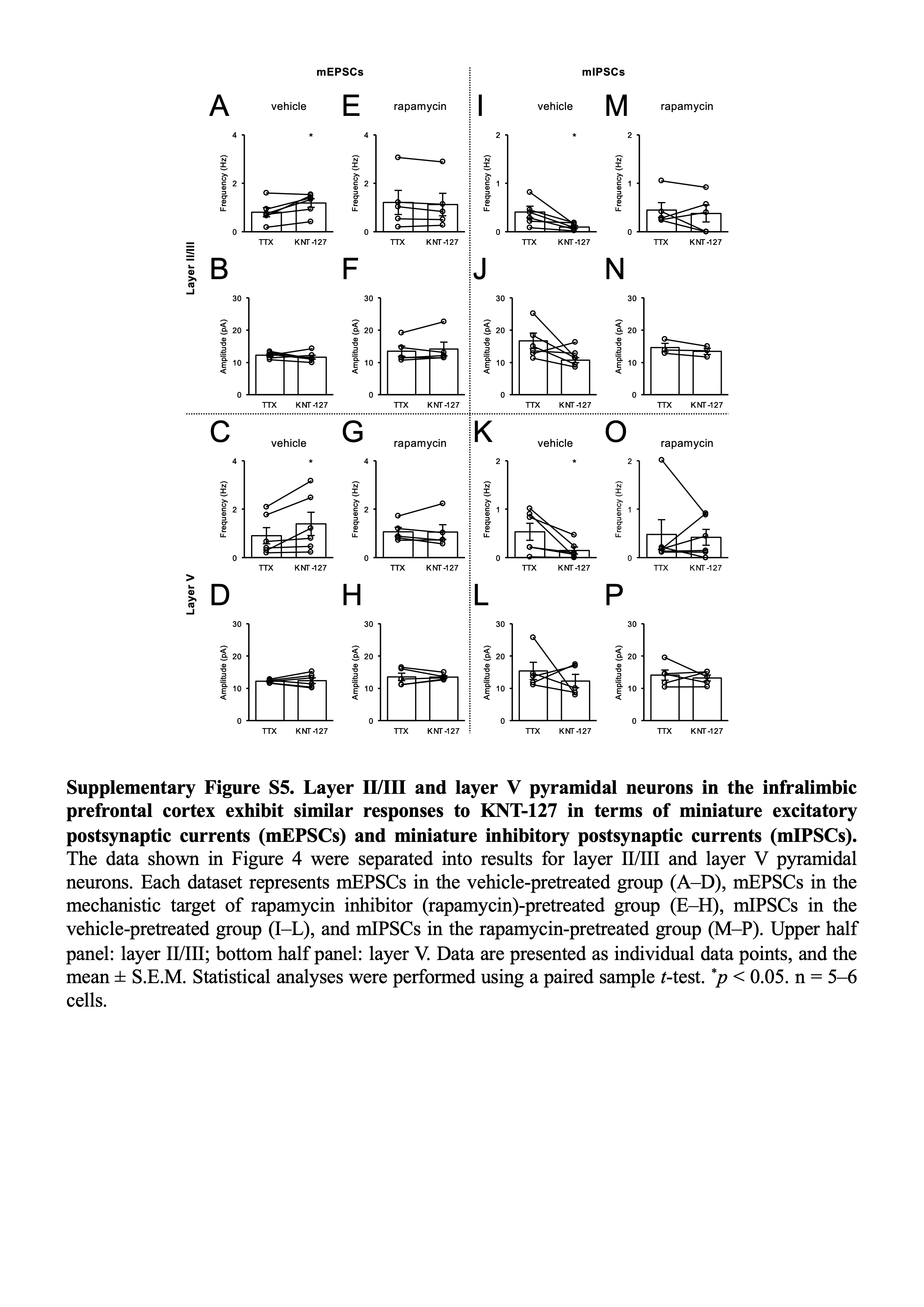

Supplement: Supplementary file 6 — Supplementary Figure S5 [file 41380_2024_2814_MOESM6_ESM.tif]
